# Autism and Visual impairment: A First Approach to a Complex Relationship

**DOI:** 10.2174/1745017902117010212

**Published:** 2021-12-22

**Authors:** Roberto Pili, Bachisio Zolo, Pericle Farris, Valentina Penna, Simona Valinotti, Gian Pietro Carrogu, Luca Gaviano, Roberta Berti, Lorenzo Pili, Donatella Rita Petretto

**Affiliations:** 1 IERFOP Onlus, University of Cagliari, Cagliari, Italy; 2 Apri Servizi, Torino, Italy; 3 Department of Education, Psychology and Philosophy, University of Cagliari, Via Is Mirrionis 1, 09127 Cagliari, Italy

**Keywords:** Autism, Blindness, Visual impairment, Diagnosis, World health organization, Psychological assessment

## Abstract

Since the first half of the 20th century there has been an interest in the study of the relationship between autism and autistic-like clinical features and with visual impairments. Autism Spectrum disorders are one of the more worldwide-studied neurodevelopmental disorder with an increasing prevalence in the last ten years. Visual impairment is a condition which derives from several causes (genetic, constitutional, injuries, nutritional and environmental ones). Again, it is a kind of spectrum and an overarching category, because visual impairments range from refractive errors (myopia, hyperopia, astigmatism), to amblyopia, strabismus, and to partial and total blindness. Since the first study of Keeler (1956) which described autistic-like patterns in five preschool children who were totally blind due to retinopathy of prematurity (ROP), a growing number of researchers addressed the relationship between autism and visual impairment. In this paper we focused on it, aiming to discuss on some lessons learned in this field and to discuss some open questions since the first research in this field.

## 1 INTRODUCTION

Autism Spectrum Disorders (ASD) are one of the more worldwide studied neurodevelopmental disorders with a neurobiological basis [1-4] and with an increasing prevalence in the last ten years [5-7]. According to recent studies, more than one child in 68 children has autism spectrum disorder, and maybe in the near future, this prevalence will increase even more [5, 6]. According to recent international diagnostic criteria, children with autism have three main features: impaired social interaction, impaired communication, and restricted and repetitive behaviors [8]. The current use of the terminology “ASD” is based on the concept of “spectrum” which, in the current psychopathology, describes the extreme heterogeneity in behavioral features in children with autism and in the range of level of impairment/s in each considered feature. According to the quality and the severity of the behavioral features and the signs and the symptoms of each individual with ASD, autism spectrum disorder has important effects on functioning and on the quality of life. Early screening path and assessment are very crucial in the promotion of specific interventions to support development. There is an increasing awareness that individuals with ASD can have some other developmental disorders (one or even more than one). Again, specific and early screening path and assessment are needed to gain information regarding the comorbidities related to different disorders and to develop specific interventions to support the developmental potential and to promote the functioning of individuals [5, 6].

In this regard, since the first half of the 20^th^ century, there has been an increasing interest in the study of the relationship between autism and autistic-like clinical features and visual impairments (VIs); there are increasing findings on the comorbidities existing between these two conditions.

“Visual impairment” (VI) is an overall clinical label that refers to different clinical conditions which could be caused by several etiologies (genetic, constitutional, lesional, nutritional and environmental ones). Again, the label “VI” refers to a kind of spectrum and an overarching category of conditions. VI impairments can range from refractive errors (myopia, hyperopia, astigmatism) to amblyopia, strabismus, and partial and total blindness; they can range from mild to severe conditions. Moreover, they can have an early or later onset in the life of individuals, and some of them can have different effects in different phases of the lifecycle (infancy, preadolescence, adolescence, adulthood).

Moreover, according to the quality and the severity of the visual impairment and the signs and the symptoms of each individual, these visual disorders can have different effects on the functioning and the quality of life of individuals. Also, for VIs, specific and early screening and assessment paths are needed to gain information regarding the clinical conditions and to develop specific interventions.

When we consider individuals with comorbidities occurring between VI and ASD, it is needless to say that specific and early screening and assessment paths are even more needed to gain information regarding comorbidities and to develop specific interventions.

In this editorial, we aim to discuss the comorbidity existing between ASD and VI with a focus on some theoretical and diagnostical problems and then discuss some lessons learned in this field; finally, we propose some open questions that need to be addressed again in the future.

### 1.1 Comorbidity Existing between Autism Spectrum Disorders and Visual Impairments

Since the first study of Keeler [9] which described autistic-like patterns in five preschool children who were totally blind due to retinopathy of prematurity (ROP), a growing number of researchers have addressed the relationship between autism and visual impairment. Soon after the study of Keeler, some other authors addressed the same topic, and this topic received increasing attention over time. In the last 30 years, two main approaches have emerged in the study of this relationship: the first approach starts from severe VI (mainly total or partial blindness) and it focuses on the prevalence of autism disorders and/or autistic-like patterns, while the second approach starts from autistic disorders and on the study of the prevalence of Vis (mainly refractive errors but also more severe visual disorders like total or partial blindness) [10-20]. In other words, in the first approach, the authors evaluated the prevalence of ASD in clinical samples of individuals with a previous diagnosis of severe VIs, while in the second approach, the authors studied the prevalence of VIs in clinical samples of individuals with a previous diagnosis of ASD.

In both approaches, a risk of under-diagnosis and under-recognition of the comorbidity between the two kinds of disorders has been described due to the diffuse tendency to focus attention on the more critical disorder and to not consider that when a child already has a neurodevelopmental disorder, there is a high risk to have another one. Unfortunately, because of this approach, there is also the tendency to not search for a second developmental disorder when a child receives a diagnosis of any developmental disorder. It is needless to underline that this approach has negative consequences not only on the diagnosis of the disorder/s but also on the cure/care, and on treatment of people with two or more neurodevelopmental disorders.

As the first approach has received more attention and it is more represented in scientific literature, in this paper, we have focused a little more on it with respect to its consequences on diagnosis and on intervention, but it is important to consider that the second approach has important effects on the same issues, too. There is an agreement that the relationship between autism and visual impairment is a close relationship; data from epidemiological studies show that there are some causes of total and partial blindness that have a closer relationship with ASD: retinopathy of prematurity (ROP), Leber’s amaurosis, optic nerve hypoplasia, septo-optic nerve dysplasia, micro-ophtalmia, anophtalmia, and CHARGE syndrome [16-19, 21-27]. According to some epidemiological studies, the relationship between these visual pathologies and ASD accounts for 30% to 90% [16-19, 21-24, 27].

But studying the relationship between VI and ASD is not a simple task, and in this field of study, there are two main topics of interest that raise questions of a theoretical nature clearly, *i.e*. diagnosis/differential diagnosis, and treatment and cure/care of people with comorbidity between ASD and VIs. In the following, we report and discuss some main points of interest related to these two topics, and we also discuss some other general points.

### 1.2 Diagnosis and Early Screening to Discover the Comorbidity between ASD and VI

Regarding diagnosis, as the conceptual models of autism have changed over the years [1, 4], there are some differences in the papers that have described and discussed the topic of diagnosis over time. In each article, the authors have used the theoretical model that was in use in that specific period, and therefore, used the current diagnostic criteria and the evaluation tools which in that particular period were considered valid and reliable for the diagnosis of autism. With reference to diagnostic criteria, since Keller's first study [9], there has been a progressive change in the terminology used to indicate autism and in the diagnostic criteria used (from DSM III to DSM-IV, and then to DSM-5) [1]. As a strictly related issue, there has been a progressive change in the use of psychometric tests for the assessment and the evaluation of the behavioral features central to and associated with ASD. In the first years of this kind of research, there was a focus on some behavioral traits, like skin picking, hand flapping, rocking. This approach was based more on the description of behavioral features than describing a diagnostical process, and the authors merely described the quantity/quality of these behavioral features. This early approach stimulated a debate because while some authors considered these behavioral traits as a consequence of blindness itself, other authors considered them as autistic-like traits [9, 28-31]. Only more recent papers have described overlapping and comorbidity between VI and ASD considering social, linguistic and communicative development in children with blindness and not only just the described behavioral patterns. These more recent papers consider the new conceptual model of autism and the authors focus assessment on the three main clinical features of autism (impaired social interaction, communication, and restricted and repetitive behavior) [8, 14, 15, 32, 33].

Due to the changing terminology used to indicate autism and the diagnostic criteria, evaluation tools and psychometric instruments for the assessment of autistic features have changed over time too. Some specific methodological problems in the diagnosis of autism in individuals with VI have emerged; while individuals with autism can have better visual and visuo-perceptual reasoning abilities than verbal reasoning abilities and linguistic abilities (*i.e*., their visual and visuo-perceptual reasoning could be a better way to assess their general abilities), individuals with both VI and ASD have added difficulties in access to visual information due to the specificity of the VI itself. As the main psychometric tools for diagnosis of autism are based on this expected neuropsychological profile and on the expected balance between strengths and difficulties of people with ASD, some of these psychometric tools (and/or some specific items in these psychometric tools) are not valid and reliable for the assessment of individuals with both VI and ASD. Recent papers have discussed and addressed those peculiar problems related to the psychometric instruments usually used for the diagnosis of autism [14, 15, 34, 35]. Moreover, considering again their verbal impairment, for individuals with ASD, access to information on a verbal basis is not possible or partially possible and it is replaced by visual access, while individuals with both VI and ASD have clear difficulties in access to verbal information and can also have communicative difficulties, but they also have difficulties in visual access. These aspects have important consequences, for example, during the complex path of diagnosis of autism in people with blindness because even visual access of information is impaired and visuo and visuoperceptual reasoning are not very simple to assess. Consequently, there is an open debate on the need to develop new psychometric instruments for the diagnosis of autism in people with blindness and/or on the need to adapt previous psychometric instruments according to these new aspects [14-16, 36]. More research is needed in this field.

### 1.3 Treatment and Cure/Care to Support the Developmental Potential of an Individual with ASD and VI

Treatment approaches and cure/care approaches to support the developmental potential of individuals with autism have changed over the years too [4]. Most of the different treatment approaches currently used for the treatment of individuals with ASD are based on visual access to information. Some specific problems adapting treatment approaches and cure/care approaches with individuals with VI and ASD have emerged, which are related to their difficulties in accessing the visual information. Recent papers have discussed and addressed those peculiar problems [16-18] and there is a general agreement on the need to develop new intervention approaches and/or to adapt previous ones to support the developmental potential of people with VI and ASD, considering the complex relationship between cognitive and neuropsychological impairments in autism and in severe VI, like blindness. Specifically, there is an agreement on the need to adapt some previous intervention approaches devoted specifically to children and adolescents with autism, considering the need to transform visual information and visual cues into tactile information and tactile cues [16-18, 37]. Again, more research is needed in this field to assess the effectiveness and the efficacy of known approaches to support the developmental and learning potential of individuals with VI and ASD and to develop and assess the effectiveness of new ones.

### 1.4 Person-centered Approach and the Role of Families

Considering the experience of people with VI and ASD, as these disorders affect all the domains of people’s life and also affect different aspects of the self and the project in life, we believe that a person-centered approach is needed involving a central role of the people with disability and of family and with an attention on their needs during all the phases of the life-cycle and on each person’s unique balance of strengths and talents [16-18].

Moreover, with a focus on the experience of families and caregivers, it is necessary to better understand their unmet needs and support them since the first phases of life of people with VI and ASD [16-18, 38]. This support is crucial since the first stages of development to gain a deeper understanding of the functional and neuropsychological profiles of the family members as a basis to develop shared ways to communicate. Again, support to families and caregivers is crucial during the intervention. Further research is needed to explore in a deeper way the unexpressed and unmet needs of caregivers, with a specific focus on communication features of people with ASD and VI, and with the aim to help caregivers and other family members to develop specific communication strategies [16, 18].

### 1.5 Transdisciplinary Approach

Regarding intervention, a transdisciplinary approach, based on the cooperation between experts in medicine, neuropsychiatry, clinical psychology, education, and other fields of mental health and rehabilitation as well as education is needed to develop evidence-based intervention and to integrate previous experience in both fields of intervention to support the developmental potential of individuals with ASD and of individuals with VI, and to increase effectiveness and efficacy for intervention devoted to the comorbidity existing between them. As a central feature of the just described person-centered approach, individuals with VI and ASD and their family have a central role in this approach and in the shared definition of short-, medium- and long-term goals.

Considering a life-long approach and a focus on daily life, the role of professionals with specific training is crucial. Educators and other professionals have a central role in supporting people with this comorbidity in every context of life (school, families, vocational sites, and other contexts) and enhancing their developmental and learning potential. A specific role is played by educators with relevant training and knowledge in both fields of typhlology and treatment of spectrum autism disorders. Their role is central in the daily-based support for people with ASD and VI, aiming to design individualized projects for school inclusion and for the development of activities of daily life and other autonomies. Moreover, these professionals also play an important role in the support of caregivers and family members.

## CONCLUSION

In this paper, we have addressed some aspects of the complex relationship between ASD and VIs. We have focused mainly on some theoretical questions that have emerged from this field of study. We also have discussed some lessons learned and some open questions related to complex themes (as diagnosis, early screening, treatment, and cure/care). Based on the literature review, we believe that a central point in this field of study is the importance to address and discover in the early phases of development the possible comorbidity exisiting between VI and ASD, as well as every kind of comorbidity between ASD and other neurodevelopmental disorders (also the ASD itself). In this paper, we have addressed some points that are specific to the comorbidity present between ASD and VI, but it is important to note that some issues considered in the paper are relevant also for Autism in general and they become even more evident in the presence of comorbidity between autism and other neurodevelopmental disorders (like those related to the need to anticipate age of diagnosis and the first intervention).

Based on the literature in this field, even if the relationship between autism and visual impairment has gained the attention of clinical professionals and researchers, only some aspects of this complex relationship are known by now, and there are some other steps that need to be taken in the near future. We believe that two different paths and approaches are needed to proceed in this field of knowledge. The first path is based on research and the second one is based on the dissemination of research results and findings. In both regards, and even more in the second one, the promotion of a network for research and clinical exchange is crucial to gain more detailed knowledge of this field and to share it. Specific training courses for health professionals are needed, together with conferences and webinars aimed to spread general knowledge and to inform public on these topics. These conferences and webinars are based on the cooperation between clinical physicians, psychologists, pedagogists, professionals in the field of education, teachers, and other health professionals.

## AUTHORS’ CONTRIBUTIONS

All the authors equally contributed to the design of the study. They have read and agreed to the published version of the manuscript.
